# Co-rumination buffers the link between social anxiety and depressive symptoms in early adolescence

**DOI:** 10.1186/s13034-017-0179-y

**Published:** 2017-08-22

**Authors:** Nejra Van Zalk, Maria Tillfors

**Affiliations:** 1 Department of Psychology, Social Work and Counselling, University of Greenwich, London, SE9 2UG UK; 2 Center for Health and Medical Psychology, JPS: Psychology, Örebro University, 701 82 Örebro, Sweden

**Keywords:** Social anxiety, Depressive symptoms, Co-rumination, Online friends, Early adolescence

## Abstract

**Objectives:**

We examined whether co-rumination with online friends buffered the link between social anxiety and depressive symptoms over time in a community sample.

**Methods:**

In a sample of 526 participants (358 girls; *M*_*age*_ = 14.05) followed at three time points, we conducted a latent cross-lagged model with social anxiety, depressive symptoms, and co-rumination, controlling for friendship stability and friendship quality, and adding a latent interaction between social anxiety and co-rumination predicting depressive symptoms.

**Results:**

Social anxiety predicted depressive symptoms, but no direct links between social anxiety and co-rumination emerged. Instead, co-rumination buffered the link between social anxiety and depressive symptoms for adolescents with higher but not lower levels of social anxiety.

**Conclusions:**

These findings indicate that co-rumination exerted a positive influence on interpersonal relationships by diminishing the influence from social anxiety on depressive symptoms over time.

## Background

During early adolescence, most youths start to spend more time with friends than with their families [7, 17], and peers become the most important source of social support [18]. Nevertheless, some peer interactions might lead to adolescents’ feeling worse rather than better in the long run. One such process is co-rumination, defined as excessive focus on problems in close dyadic relationships with peers [44]. A co-ruminating relationship features frequently discussing problems, mutual encouragement of discussing problems, discussing the same problems repeatedly, focusing on negative feelings, and speculating about problems in general [44]. A large and ever-growing literature indicates that co-rumination might result in increases in depressive symptoms in adolescence [3, 23, 44, 54, 58]. Nevertheless, even though co-rumination might help perpetuate issues such as depressive symptoms over time, it is also linked to positive friendship quality and emotional closeness [44], offering a supportive interpersonal context for adolescents. Indeed, different aspects of co-rumination, such as extensively talking about problems, has been linked with positive friendship adjustment in adolescence [46], and co-rumination is not necessarily detrimental in high-quality relationships in emerging adulthood [34]. Thus, co-rumination might have both negative and positive influences in the context of friendships.

### The links between co-rumination, social anxiety, and depressive symptoms

Forming and maintaining friendships in the first place is not easy for all adolescents, however, and the effects of co-rumination on the development of depressive symptoms have not been thoroughly tested regarding social anxiety. Non-clinical social anxiety is categorized by social fears, excessive discomfort, rumination, and somatic symptoms such as trembling, blushing and sweating before, during, and after social interactions [24]. Numerous studies show a persistent link between social anxiety and non-clinical depressive symptoms from childhood throughout adulthood (e.g., [26, 31, 66]). In this study, we propose that co-rumination with peers might be less maladaptive for socially anxious adolescents because it might buffer the link between social anxiety and depressive symptoms. Socially anxious adolescents tend to have difficulties forming friendships [61], and social anxiety affects perceptions of support and intimacy [30] as well as acceptance into peer groups [20] in a negative way. According to Cohen and Wills’ classical definition of buffering [10], support is associated with well-being for individuals who are under stress, because it buffers potentially negative influences of stressful events. In this case stress refers to social anxiety. For socially anxious adolescents, then, being able to frequently discuss problems with friends might have another consequence, as it could boost their social skills and help relieve their sense of loneliness, thereby resulting in less depressive symptoms over time. As lack of social support might be a risk factor for developing symptoms of depression for shy adolescents [36], this seems a reasonable assumption. However overindulgent a co-ruminating relationship might be for adolescents who do not struggle with social anxiety, we propose that it might buffer the links with depressive symptoms for those who do.

To our knowledge, only a handful of studies have investigated the links between social anxiety, depressive symptoms, and co-rumination, albeit not directly, and with mixed results. One study with 83 nearly 14-year-old girls showed that co-rumination was negatively related to social anxiety when controlling for depressive symptoms, so that girls with more social anxiety co-ruminated less with their friends [51]. The authors believe these results to be expected, as socially anxious individuals usually have fewer opportunities to co-ruminate due to having fewer close friends overall [30], and are less likely to self-disclose to others [1]. Nevertheless, these links were only investigated cross-sectionally rather than longitudinally, and only for girls. The question thus still remains what these links would look like over time. A three-wave longitudinal study showed that social anxiety indirectly predicted higher levels of co-rumination via rumination over time for girls [25]. This study did not explore depressive symptoms per se, however. A reason for these mixed findings in the current literature could be that rather than being a direct predictor, co-rumination might instead moderate the link between social anxiety and depressive symptoms. That is, via unwarranted focus on problematic issues with close peers, co-rumination might buffer the effect of social anxiety on depressive symptoms. Nonetheless, an examination of the buffering effects of co-rumination on the link between social anxiety and depressive symptoms still awaits testing.

### The context of online friendships

In addition, most studies examining the effects of friendships on adolescent well-being are focused on real-life, or offline friends—often school peers. There are reasons to believe that online friendships might be just as important for adolescent adjustment, however, especially for socially anxious adolescents. Highly socially anxious youths tend to be victimized by offline peers [13, 16, 30, 31, 50, 55], and are often lonely [53]. Communicating with significant friends online might be a viable option to an unsatisfactory or maybe even non-existing social network in socially anxious adolescents’ everyday lives. Indeed, according to the *social compensation* hypothesis, socially anxious adolescents are believed to be drawn to the Internet to compensate for their anxieties during offline social interactions [2]. Making friends online might be particularly advantageous for youths high in social anxiety, because it is likely easier compared to offline [32, 47]. Online communication is particularly appealing over face-to-face interactions to socially anxious adolescents because of factors such as enhanced controllability of self-presentation as well as self-disclosure [48, 59, 64]. Having an online confidant to discuss problems with might have positive effects on socially anxious youth’s well-being, despite the co-ruminating elements. Whether co-rumination with close online peers might buffer the links between social anxiety and depressive symptoms has, to our knowledge, never been directly tested.

### Aims and hypotheses

In this study, we attempt to fill the gaps in the current literature about the effects of co-rumination with close online friends on the links between non-clinical social anxiety and depressive symptoms. We focus on online friendships, which are easier to obtain compared to offline friendships for adolescents with high levels of social anxiety. We use a longitudinal community-based sample of 526 participants (358 girls; *M*_*age*_ = 14.05) followed for 16 months. Early adolescence appears to be the time of onset for non-clinical social anxiety [42] as well as depressive symptoms [43], and friendships become increasingly important during the transition from middle childhood to adolescence [6], making this developmental period of particular significance to explore. Typically speaking, social anxiety has been found to precede depressive symptoms [42]. However, depressive symptoms might also affect how social anxiety develops, making direction of causality important to address. We therefore explore bidirectional effects between all main study constructs. In addition, an important characteristic pertaining to dyadic friendships is friendship stability, as higher levels of depressive symptoms in adolescence are linked with lower friendship stability [40]. As early adolescence is generally marked by unstable friendships (e.g., [28, 49]), and friendship stability might matter in terms of the processes in focus, we control for friendship stability in all analyses. Because friendship quality is linked to both decreases in social anxiety [14] as well as increases in co-rumination [45], we control for friendship quality over time as well. As a next step, we add latent interactions between Time-1 co-rumination and social anxiety predicting Time-2 and Time-3 depressive symptoms, respectively. In line with previous findings, we expect that (I) social anxiety and co-rumination will predict depressive symptoms over time, and that (II) depressive symptoms will in turn predict both social anxiety as well as co-rumination. In terms of buffering effects, we expect that (III) co-rumination will moderate the link between social anxiety and depressive symptoms, but only for adolescents with higher levels of social anxiety.

## Methods

### Sample

Participants were adolescents roughly aged 13–15 from a medium-sized town in Sweden (with a population of about 135,000), who took part in a three-wave longitudinal study. The data collections took place in school and online with approximately 8 months between each time point. The first data collection took place in September 2010 (Time 1), followed by the second measurement in May 2011 (Time 2) and a final measurement in January 2012 (Time 3). We initially recruited 423 adolescents (205 girls; *M*_*age*_ = 14.05) comprised of 7th–9th graders from one school. The participants were evenly distributed across three classrooms per each grade. Approximately 12.1% of all participants were first-generation immigrants at the onset of the study, which was slightly lower compared to 14.7% in the entire country, according to official reports [57]. The unemployment rate (6%) and the proportion of single-parent households in the community (5.1%) were similar to the rest of the country [57]. Mean incomes were about 5% lower compared to the rest of Sweden [57].

The initial participants were in turn asked to invite three close online (not real-life or offline) friends with whom they had frequent contact with to take part in the study. The online friends were defined as someone the adolescents had regular rather than occasional interactions with in online settings only, who was a very important person in their life, somebody to talk to, spend time with, and do things with [27]. In addition, they could live anywhere, did not have to be of the same age, and could be a boy or a girl. The online friends could not be a parent or an adult similar to a parent, nor a sibling, however. The average number invited by the original participants was .13 for Time 1, .39 for Time 2, and .40 for Time 3. The majority of the online relationships were same-sex relationships (65–73% at Time 1; 65–79% at Time 2, and 69–78% at Time 3). All of the online friends were in turn invited to take part in the study, with 103 adolescents choosing to participate overall. The online friends needed to confirm the relationship they had with the target adolescents in order to be included in the study. Information about how long they had been friends online was not collected, however. Together with the initial sample, then, the final target sample thus comprised 526 13–15 year olds (358 girls; *M*_*age*_ = 14.05), with 72% of the adolescents reporting data on all study variables at all three time points.

### Procedure

Before the data collection took place, parents were informed about the study through a meeting at the school, to which they were invited by the schoolteachers. Passive consent was used, as the parents received a pre-paid post card to return to us if they did not want their child to participate (only 2% of the parents did so). The parents were informed that they could withdraw their child from the study at any time.

For the initial sample, information was collected through a combination of offline and online questionnaires at Times 1 and 2, and then via online-only questionnaires at Time 3. The reasoning behind a combination of online and offline questionnaires at Times 1 and 2 was to shorten the time the participants had to spend filling out the information in school, as well as to separate the items referring to offline and online activities. The online questionnaires were thus focused on detailed questions about adolescents’ online-exclusive activities and friendships. In this way, we prevented problems that previous studies faced, where adolescents were asked to recall information about friends in another context (e.g., [56]). At Time 3, however, participants who were 9th graders at Time 2 had started high school and changed schools. In order to retain as many participants in the sample as possible, as well as to keep the goodwill of the school principal and the staff, we decided to conduct the 3rd wave data collection online-only for everyone involved. In addition, online questionnaires were reported to be the preferred method of data collection by the participants. There were no differences on the main study variables across the time points between the participants who filled out questionnaires online and offline, versus online-only.

During the in-school data collection, trained research assistants visited the adolescents in their classrooms during school time. The teachers were not present. The adolescents were informed about the types of questions they would answer, and the time it would take to finish the questionnaires. They were also informed that their participation was voluntary, and that if they chose not to participate, they could do something else instead. They were guaranteed that if they did participate in the study, their answers would never be shown to anyone. After the adolescents filled out the offline questionnaire at school, they were instructed to fill out the second part online (all of the adolescents did so). To complete the online questionnaire, the adolescents were sent an e-mail including a username, password and a link to the questionnaire itself. They filled out the online part of the questionnaire in their own time. They reported each online friend’s first and last name, gender, age, and e-mail address, and the nominated friends were e-mailed a participation link in turn.

For the online friends who were invited to take part via the snowballing method, the procedure for the data collection was identical to that of the initial targets, with the exception that they filled out all of the questionnaires online at all three timepoints. The consent for the invited friends’ data collection was achieved in the same way as for the initial targets, except that the parents weren’t initially contacted via meetings at school, but received information about the study directly through post. Thus, just like for the initial targets, the parents contacted us if they didn’t wish their child to partake in the study (none of the parents for the online-only subsample did so). No participant was paid for taking part in the study; however, all participants received two gift cards for cinema tickets. The Regional Ethics Committee approved the procedures and measures used in the study.

### Measures

The means and descriptives for all study variables are shown in Table 1. The Cronbach’s alphas refer to raw rather than latent measures. For the initial sample, measures about social anxiety and depressive symptoms were collected offline at Times 1 and 2, whereas the other measures were collected via the online survey. At Time 3, all measures were collected online. For the online subsample, however, all measures were collected online at all timepoints.

**Table 1:** Means (SDs) and correlations for all study variables

	Mean (SD)	1	2	3	4	5	6	7	8	9	10	11	12	13
1. Social anxiety T1	1.38 (.30)	–												
2. Social anxiety T2	1.36 (.33)	.73***	–											
3. Social anxiety T3	1.47 (.40)	.61***	.64***	–										
4. Co-rumination T1	3.21 (1.25)	.03	.07	.07	–									
5. Co-rumination T2	3.22 (1.08)	.03	.05	.04	.64***	–								
6. Co-rumination T3	3.12 (1.29)	.11	.11	.08	.42***	.52***								
7. Depressive Sym. T1	1.86 (.58)	.24***	.24***	.17***	.08	.02	.10	–						
8. Depressive Sym. T2	1.84 (.62)	.25***	.30***	.24***	.09	.06	.17*	.66***	–					
9. Depressive Sym. T3	2.00 (.68)	.37***	.34***	.48***	.06	−.01	.13*	.52***	.56***	–				
10. Friendship Qual. T1	4.11 (.93)	.02	.06	.03	.59***	.41***	.30***	.05	.13*	.01	–			
11. Friendship Qual. T2	4.18 (.87)	.05	.05	−.05	.43***	.58***	.35***	−.04	−.01	−.09	.47***	–		
12. Friendship Qual. T3	4.31 (.77)	−.04	−.06	−.11	.32***	.39***	.53***	−.02	.03	−.07	.34***	.42***	–	
13. Friendship Stab. T1	1.24 (.43)	.04		.03	.11*	.07	.07	−.05	.03	−.08	.14*	.09	.04	–

* p < .05

** *p* < .01

*** *p* < .001

#### Social anxiety

Non-clinical social anxiety was measured with questions about fears in different social situations [21]. This instrument is a modified version of the Social Phobia Screening Questionnaire, which was originally created for adults [19] and adjusted for children and adolescents up to age 18 (the SPSQ-C, or the Social Phobia Screening Questionnaire for Children; [21]). The instrument measures 8 social situations that tend to elicit social anxiety, such as “speaking in front of the class,” “going to a party,” and “being with classmates during breaks.” Adolescents rated their fears on a three-point scale ranging from *None* (1), *Some* (2), to *A lot* (3). The Cronbach’s alpha was .72 for Time 1, .78 for Time 2, and .84 for Time 3.

#### Co-rumination with best online friend

Eight questions about co-rumination were used from the revised co-rumination questionnaire [62]. The original revised version used questions about adolescents’ co-rumination about their problems with their mothers. In this study, we instead measured how the target participants talk about their problems with their best friends. The items measured to what extent the adolescents typically co-ruminated about when they have a problem and how they and their best friend usually talk about it. Examples of items were: When I have a problem, “my friend and I talk to each other about it for a long time,” “we’ll talk about every part of the problem over and over,” and “we talk a lot about all of the different bad things that might happen because of the problem.” The response items were (1) *Not at all true,* (2) *A little true*, (3) *Somewhat true*, (4) *Mostly true,* and (5) *Really true*. The Cronbach’s alpha for this scale was .95 for Time 1, .96 for Time 2, and .95 for Time 3.

#### Depressive symptoms

Depressive symptoms were measured with a shortened version of the Child Depression Scale from the Center for Epidemiological Studies (the CESD-10; [41]), which assesses depressive symptoms such as worry, sadness, hopelessness, lethargy, and poor appetite [8]. The shortened version includes 10 questions based on a factor analysis conducted on the original 20-item scale, and gauges non-clinical symptoms. The response items were *Not at all* (1), *Occasionally* (2), *From time to time* (3), and *Often* (4). Participants were instructed to think about the past week. Examples of items were: “How often have you worried about things you don’t usually worry about,” “How often have you felt down and unhappy,” and “How often have you felt sad?” The Cronbach’s alpha was .81 for Time 1, .85 for Time 2, and .88 for Time 3.

#### Control variables

##### Friendship quality with best online friend

Adolescents were asked to think about the very best friend they had nominated (the 1st on their list of nominations). They were then asked about the quality of the friendship, as indicated by 6 questions about perceived support and trust based on Parker and Asher’s well-used scale [38]. Examples of items were: “My friend supports me when I have an argument with my parents/teachers,” “My friend pays attention to my feelings,” and “My friend stands by me when others talk about me behind my back.” The response items were *Not at all true* (1), *A little true* (2), *Somewhat true* (3), *Pretty true* (4), and *Really true* (5). The Cronbach’s alphas were .90 for Time 1, and .91 for Times 2 and 3.

##### Friendship stability with best online friend

The data collected for 1st best online friend was re-coded to indicate friendship stability across time. For those who reported no stable friendships across any of the time points, the stability variable was coded as 0. For those who reported the same friend from one time point to another, as well as across all three time points, the variable was re-coded as 1. There were 175 adolescents who reported stable friendships either from Time 1 to Time 2, from Time 2 to Time 3, or across all three time points. Three hundred and fifty-one adolescents did not report stable friendships. These numbers are similar to those found in many other longitudinal studies on early adolescents [28, 49], indicating that early adolescence in general is a period of unstable friendships.

### Plan for analyses

Using the MPlus 7.0 software [37] with the full information maximum likelihood (FIML) procedure for all analyses, we conducted a series of structural equation models (SEM) to assess directional associations between social anxiety, depressive symptoms, and co-rumination with online best friend, controlling for friendship quality and friendship stability with best online friend. Creating latent variables allowed us to estimate constructs minimizing measurement error, avoid regression to the mean, and examine the direction of causality between all study variables [33]. In addition, when estimating latent interactions, the shared variance (or within-time co-variance) between the outcome variables at Times 2 and 3 is taken into account. Hence, by modeling latent rather than observed interactions (using the XWITH command) allowed us to minimize the measurement error from these independent predictors, which resulted in a more precise estimation of the interaction effect [35]. We used the following indices of model fit: the root mean square error of approximation (RMSEA) [5] and the comparative fit index (CFI) [4]. RMSEA values of less than .08 represent an acceptable fit, whereas values less than .05 are considered a very good fit [5]. CFI values above .95 are considered acceptable fit, whereas values greater than .97 are considered good fit [4].

#### Identifying the longitudinal CFA model

Before being entered into the analyses, the items for social anxiety, co-rumination, depressive symptoms, and friendship quality were parceled by averaging the scores with the fixed-factor method of scaling, where the latent variance was fixed at 1 and the latent mean was fixed at 0 (as recommended by [33]). The factor loadings for the parcels ranged from .60–.82 for social anxiety, .80–.90 for depressive symptoms, .88–.96 for co-rumination, and .82–.89 for friendship quality at all three time points (*p* < .001).

We then tested whether the longitudinal constructs were the same across all measurement occasions. The different levels of invariance are supported if the changes in model fit from a lower to a higher level of invariance are minor [9, 33]. The results are shown in Table 2. First, we identified an independence null model (according to [33]). This null model assumes no expectations of change in the variances or the means of our constructs across all time points. As would be expected, the null model had a poor fit. Second, we fitted a configural-invariant model to the data, where the expected pattern of loadings were specified at each time point, and the residual variances were correlated across all time points [33]. As Table 2 shows, this model had an appropriate fit. Third, we fitted a weak factorial invariance model to our data, where the loadings of each indicator were set to be equal across the time points [33]. This model showed an acceptable fit, which indicates that that the longitudinal constructs used in our baseline model were the same across the three time points. In addition, the CFI did not increase with more than .01, which is considered to support the level of invariance [9, 33]. Finally, we fitted a strong invariance model, where all intercepts are specified to be equal across time points [33]. The change in CFI was not acceptable, and we thus used the weak invariance model for further testing.

**Table 2:** Model fit statistics for tests of invariance with social anxiety, depressive symptoms, co-rumination, and friendship quality across the three time points

Model tested	χ^2^	*df*	*p*	∆χ^2^	∆*df*	*p*	RMSEA	RMSEA 90% CI	CFI	∆CFI	NNFI/TLI	∆TLI	Pass?
Null model^a^	11463.105	888	<.001	–		–	.157	–	.090	–	.118	–	No
Measurement model estimates													
Configural invariance	1111.037	711	<.001	–	–	–	.034	0.030/0.038	.966	–	.958	–	Yes
Weak invariance	1132.180	731	<.001	21.143	20	–	.034	0.030/0.037	.965	.000	.959	.001	Yes
Strong invariance	1493.899	753	<.001	361.719	22	–	.045	0.042/0.048	.936	.029	.927	.032	No
Latent model estimates													
Omnibus test^b^	1212.815	750	<.001	281.084	3	–	.036	0.032/0.039	.960	.024	.954	.027	Yes

^a^Independence null model

^b^Including within-time variances, co-variances, and cross-lagged paths

#### Adding the structural relationships

After thus identifying the measurement model, we added structural relationships between the latent variables. The stabilities, cross-lagged paths, and within-time co-variances between all the constructs at each time point were added to the model. The results from this omnibus test are shown in the lowest part of Table 2. The model showed an improvement in fit, and was thus deemed acceptable. This model was then used in all subsequent analyses as a baseline model.

## Results

### The longitudinal links between early adolescent social anxiety, depressive symptoms, and co-rumination with best online friends

**Fig. 1:**
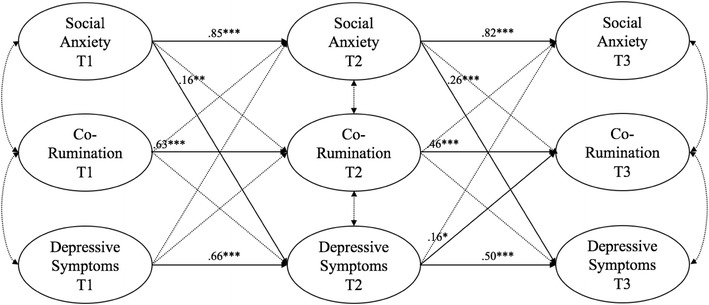
Links between main study variables at the three time points, controlling for friendship stability and friendship quality. For the sake of clarity, non-significant paths are *dashed*, and control variables are omitted from the figure. *p < .05, **p < .01, ***p < .001

We began by adding Time-1 friendship stability as a covariate to the final baseline model, allowing associations with all other main study constructs at all three time points. In this way, we excluded the possibility of friendships being more or less stable interfering with our results. This model is illustrated in Fig. 1. For the sake of clarity, only significant paths are shown in the figure, and the control variables are excluded due to complexity. All standardized paths are shown in Table 3. This model had a good fit (χ^2^ = 1383.37; *df* = 784; *p* < .0001; RMSEA = .04; CFI = .95). As can be seen in Fig. 1, social anxiety predicted an increase in depressive symptoms at both time points, but not vice versa. Depressive symptoms at Time 2 predicted an increase in co-rumination at Time 3, but not the other way around. Nevertheless, no direct links between social anxiety and co-rumination emerged in this model, and no bidirectional links between depressive symptoms and social anxiety were found.

**Table 3:** Standardized results for main model

	β
Predicting social anxiety at time 2	
Social anxiety T1	.85***
Depressive symptoms T1	.002
Co-rumination T1	.03
Friendship quality T1	.04
Friendship stability T1	.04
Predicting social anxiety at time 3	
Social anxiety T2	.82***
Depressive symptoms T2	−.08
Co-rumination T2	.05
Friendship quality T2	−.15*
Friendship stability T1	−.03
Predicting co-rumination at time 2	
Co-rumination T1	.63***
Social anxiety T1	.10
Depressive symptoms T1	−.05
Friendship quality T1	.13
Friendship stability T1	.09*
Predicting co-rumination at time 3	
Co-rumination T2	.46***
Social anxiety T2	−.02
Depressive symptoms T2	.16*
Friendship quality T2	.14
Friendship stability T1	.03
Predicting depressive symptoms at time 2	
Depressive symptoms T1	.66***
Social anxiety T1	.16**
Co-rumination T1	−.02
Friendship quality T1	.16**
Friendship stability T1	.02
Predicting depressive symptoms at time 3	
Depressive symptoms T2	.50***
Social anxiety T2	.26***
Co-rumination T2	.02
Friendship quality T2	−.09
Friendship stability T1	−.09
Predicting friendship quality at time 2	
Friendship quality T1	.40***
Social anxiety T1	.12
Co-rumination T1	.28***
Depressive symptoms T1	−.11
Friendship stability T1	.02
Predicting friendship quality at time 3	
Friendship quality T2	.33***
Social anxiety T2	−.17*
Co-rumination T2	.27***
Depressive symptoms T2	.09
Friendship stability T1	.01

* p < .05

** p < .01

*** p < .001

### Does co-rumination with best online friends buffer the link between social anxiety and depressive symptoms?

We added a latent interaction between social anxiety and co-rumination at Time 1 to the latter baseline model controlling for friendship stability and friendship quality. We used the latent moderated structural equations (or XWITH) approach for investigating the interaction [29]. Using the interaction at Time 1, we predicted depressive symptoms at Times 2 and 3, respectively, according to recommendations about using latent interactions [33]. In order to exclude the possibility of co-rumination being a moderator between depressive symptoms and social anxiety rather than the other way around, we also tested the reverse interaction between Time-1 depressive symptoms and co-rumination predicting social anxiety. Due to the nature of the procedure, no model fit statistics are given, and the interaction effects are unstandardized.

**Fig. 2:**
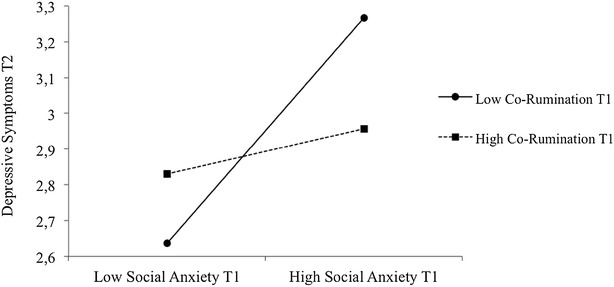
Two-way latent interaction effects for Time-1 social anxiety and co-rumination predicting Time-2 depressive symptoms. *High* is indicated by 1 SD above, whereas *low* is 1 SD below the mean, respectively

The only significant interaction that emerged was between Time-1 social anxiety and co-rumination predicting Time-2 depressive symptoms (latent unstandardized estimate = −.13; *p* < .05). We plotted this interaction by using the two-way interaction effects for unstandardized variables, with depressive symptoms at Time 2 as the outcome (controlling for the effects of Time-1 depressive symptoms), Time-1 social anxiety as the predictor, and Time-1 co-rumination with best friend as the moderator. We used 1 SD above and below the mean when probing the interaction, which is depicted in Fig. 2. As is shown in the figure, adolescents with the combination of high social anxiety and low co-rumination with best friend at Time 1 had the highest levels of Time-2 depressive symptoms. This was not the case for the combination of Time-1 high co-rumination and high social anxiety, as it predicted lower depressive symptoms at Time 2. In addition, the combination of low social anxiety and low co-rumination at Time-1 predicted the lowest depressive symptoms at Time 2. According to our expectations, then, these results indicate that high co-rumination buffers depressive symptoms for adolescents with high, but not low social anxiety.

## Discussion

Non-clinical social anxiety has been linked to depressive symptoms from childhood throughout adulthood (e.g., [26, 31, 66]). In this study, we focused on the process of co-rumination with close online friends, and how it might contribute to the development of depressive symptoms over time. In order to test the overall links between social anxiety, depressive symptoms, and co-rumination, we controlled for the effects of friendship stability and friendship quality, and used a latent cross-lagged model across three time points. In line with our first hypothesis, our results indicate that social anxiety predicts depressive symptoms at both time points. Contrary to our second hypothesis, however, depressive symptoms were not a significant predictor of either social anxiety or co-rumination over time. Instead, we found significant moderating effects of co-rumination on the link between social anxiety and depressive symptoms. In confirmation of our third hypothesis, co-rumination buffered the link between social anxiety and depressive symptoms, but only for adolescents with high levels of social anxiety. Previous research on the impact of co-rumination on the link between social anxiety and depressive symptoms is scarce, with only a few studies indirectly focusing on the issue. The findings from the current study support the notion that socially anxious adolescents’ co-ruminating online interactions might buffer the development of depressive symptoms over time, over and above other friendship effects.

Only a handful of studies have previously focused indirectly on the current topic. One cross-sectional study indicated that co-rumination was negatively related to social anxiety when controlling for depressive symptoms, so that girls with more social anxiety co-ruminated less with their friends [51]. As socially anxious individuals usually have fewer opportunities to co-ruminate due to having fewer close friends overall [30], and are less likely to self-disclose to others [1], the authors believed these findings to be anticipated. Nevertheless, such links might be different for online friends, as socially anxious adolescents appear to benefit from such friendships compared to youths without social anxiety. Our findings indicate no concurrent or prospective links between social anxiety and co-rumination, however. The way co-rumination was measured in this study was within the context of close online friendships, with adolescents reporting on how much they co-ruminated with the online friend they already had. In another study, girls’ social anxiety indirectly predicted higher levels of co-rumination via rumination [25]. Nevertheless, depressive symptoms, albeit related to rumination, were not directly explored. In addition, none of these studies have examined co-rumination as a potential moderator. To our knowledge, this is the first study to examine buffering effects of co-rumination on the links between social anxiety and depressive symptoms within the context of online friendships. We believe that an explanation for these findings lies in perceptions of increased closeness and social support in online relationships. Our findings partially support this notion, as there were positive associations between co-rumination and friendship quality.

Because we looked at online friendships, this might help explain the discrepancy in results compared to some previous studies. Online communication is particularly appealing over face-to-face interactions for socially anxious adolescents, because of factors such as enhanced controllability of self-presentation and self-disclosure [48, 59, 64]. Studies indicate that making friends online might be particularly advantageous for highly socially anxious youths, because it is easier compared to offline [32, 47]. Indeed, shy adolescents’ self-esteem is predicted by having exclusively online friends, which in turn predicts forming more friendships online as well as offline [60]. These results support the social compensation hypothesis, which states that socially anxious adolescents use online communication to compensate for social inadequacies during offline interactions [2]. In this study, adolescents with high levels of social anxiety who reported higher levels of co-rumination with close online peers also had the lowest levels of depressive symptoms over time. Another related reason for the current findings might be that by discussing problems with friends, socially anxious individuals gain better social skills. Social anxiety is associated with not being able to utilize social skills due to anxiety, as well as failure during social interactions [11, 52]. Spending time with others enhances social skills, however, but socially anxious individuals tend to avoid social interactions in general [11]. In addition, when adolescents lack communication with peer groups they appear to co-ruminate with close friends in order to better understand their problems, suggesting that co-rumination is likely a coping strategy for depressive symptoms due to feeling lonely [12]. For socially anxious adolescents, then, mere exposure to social interactions might be sufficient in buffering depressive symptoms over time by helping them feel less lonely and increasing friendship quality.

Typically speaking, co-rumination is measured as interpersonal interaction with a close same-sex friend, but the concept has been extended beyond adolescent friendships to college roommates [34], adult coworkers [22], and even mother–child dyads [62, 63]. To our knowledge, however, there are no studies focusing on how online interactions with close friends might impact co-rumination. As we have argued previously, however, there are reasons to believe that this type of interaction is of importance, especially for early adolescents. Swedish data from the time of the current data collection indicated that 90% of early adolescents used the Internet daily, with boys spending around 19.1 h and girls 14.2 h online at home every week [15]. Studies also show that online friends have just as much significance in young people’s lives compared to offline friends, especially for socially anxious adolescents, as they might miss out on offline interactions due to their anxiety and thus turn to the Internet to find friends there [2, 39, 65]. Taking into account online interpersonal communication is therefore of particular interest when looking at processes associated with close relationships, such as co-rumination.

The current study has some limitations. First, we used data with approximately 8 months between the time lags, which aren’t necessarily the most appropriate lags in terms of the ability to detect associations between the constructs used in the study. The changes between the variables might happen either at a faster or a slower pace than the 8-month measurement points used in the current study. Another limitation is the sole use of self-reports, which could result in the problem of shared method variance. Nonetheless, as the latent interaction used in our model removes co-variation between the variables during the modeling procedure, the results cannot be attributed to common variance. This study also only assessed one type of communication (i.e., chatting online), whereas adolescents might use other forms of online communication as well, such as the use of video and audio. Nevertheless, at the time of the data collection, chatting using computers was the most common way of acquiring new friends, which transpired from our pilot testing and is the reason why we limited the options to measure this way of communicating. In addition, the fact that the measures were collected in-school and online at Times 1 and 2, but online-only at Time 3, might have impacted the results. Nevertheless, creating latent constructs in SEM helps to reduce measurement error [33]. We also achieved invariance when testing the stability of our constructs, thus indicating that our constructs were stable over time. Finally, the current results refer to non-clinical levels of social anxiety as well as depressive symptoms, and can thus not be generalized to clinical populations. Despite its limitations, however, the current study has several strengths. We have used a three-wave longitudinal sample of early adolescents followed over time, and we have analyzed our results using autoregressive cross-lagged models—allowing us to minimize measurement error and investigate bidirectional processes. We have also used a representative community sample of early adolescents living in a middle-sized community in Sweden. Thus, our findings offer one novel explanation as to how the link between early adolescents’ social anxiety and depressive symptoms might be buffered by co-ruminating with online peers.

Young people face many challenges on a daily basis, and for those with difficulties to interact with others in social contexts such as socially anxious adolescents, early adolescence is likely a very stressful time. A large body of research shows a persevering link between non-clinical social anxiety and depressive symptoms throughout adolescence and adulthood. Our results indicate that in order to improve the understanding about the associations between social anxiety and depressive symptoms, it is important to look beyond the individual and into young people’s surrounding social contexts as well—including those online. The findings from this study support the notion that co-rumination is by its very nature a complex construct, as it might represent both adaptive as well as maladaptive aspects, which in turn reflects the intricacy of social interactions [44]. These intricacies are ever increasing with online peers becoming more important for adolescents’ daily interactions. Despite the maladaptive aspects of online co-rumination, such as repetitive chatting about negative events and feelings, validation by close online peers might nevertheless help foster an adaptive buffering effect over time, especially for socially anxious adolescents who seldom self-disclose in general. Hence, understanding how the use of social interaction strategies such as online co-rumination might affect the severity of social anxiety in particular might subsequently aid in understanding how to prevent the development of depressive symptoms and other co-morbid emotional problems at an early stage.
